# An Environmental Scan of Sex and Gender in Electronic Health Records: Analysis of Public Information Sources

**DOI:** 10.2196/20050

**Published:** 2020-11-11

**Authors:** Francis Lau, Marcy Antonio, Kelly Davison, Roz Queen, Katie Bryski

**Affiliations:** 1 School of Health Information Science University of Victoria Victoria, BC Canada; 2 Canada Health Infoway Toronto, ON Canada

**Keywords:** sex, gender, electronic health records, standards, transgender persons

## Abstract

**Background:**

Historically, the terms sex and gender have been used interchangeably as a binary attribute to describe a person as male or female, even though there is growing recognition that sex and gender are distinct concepts. The lack of sex and gender delineation in electronic health records (EHRs) may be perpetuating the inequities experienced by the transgender and gender nonbinary (TGNB) populations.

**Objective:**

This study aims to conduct an environmental scan to understand how sex and gender are defined and implemented in existing Canadian EHRs and current international health information standards.

**Methods:**

We examined public information sources on sex and gender definitions in existing Canadian EHRs and international standards communities. Definitions refer to data element names, code systems, and value sets in the descriptions of EHRs and standards. The study was built on an earlier environment scan by Canada Health Infoway, supplemented with sex and gender definitions from international standards communities. For the analysis, we examined the definitions for clarity, consistency, and accuracy. We also received feedback from a virtual community interested in sex-gender EHR issues.

**Results:**

The information sources consisted of public website descriptions of 52 databases and 55 data standards from 12 Canadian entities and 10 standards communities. There are variations in the definition and implementation of sex and gender in Canadian EHRs and international health information standards. There is a lack of clarity in some sex and gender concepts. There is inconsistency in the data element names, code systems, and value sets used to represent sex and gender concepts across EHRs. The appropriateness and adequacy of some value options are questioned as our societal understanding of sexual health evolves. Outdated value options raise concerns about current EHRs supporting the provision of culturally competent, safe, and affirmative health care. The limited options also perpetuate the inequities faced by the TGNB populations. The expanded sex and gender definitions from leading Canadian organizations and international standards communities have brought challenges in how to migrate these definitions into existing EHRs. We proposed 6 high-level actions, which are to articulate the need for this work, reach consensus on sex and gender concepts, reach consensus on expanded sex and gender definitions in EHRs, develop a coordinated action plan, embrace EHR change from socio-organizational and technical aspects to ensure success, and demonstrate the benefits in tangible terms.

**Conclusions:**

There are variations in sex and gender concepts across Canadian EHRs and the health information standards that support them. Although there are efforts to modernize sex and gender concept definitions, we need decisive and coordinated actions to ensure clarity, consistency, and competency in the definition and implementation of sex and gender concepts in EHRs. This work has implications for addressing the inequities of TGNB populations in Canada.

## Introduction

### Background

The collection of sex and gender data as part of a person’s demographic information in electronic health records (EHRs) has been in place for decades [1]. Historically, the 2 terms have been used interchangeably as a binary attribute to describe an individual as being male or female. There has been little effort to define this attribute and its values consistently across EHRs, health care organizations, and health information standards [2]. Longstanding use of poorly understood and defined sex and gender concepts has created much systemic inconsistencies. Different EHRs use either sex or gender as the data element name for this attribute and a wide range of coding schemes with letters and numbers such as *F*, *M*, *1*, *2*, *U*, and *UNK* to represent the values [2-4]. There is also growing recognition that sex and gender are distinct concepts [5]. Sex is a biological construct based on anatomy, genetics, and hormones that distinguish between males and females on a continuum. Gender is a psychological and social construct based on attitudes, feelings, behaviors, and cultural factors a person may use to identify and express their authentic identity as a man, woman, or other options. Given the diversity that exists in our society, it is inaccurate, inadequate, and inappropriate to use a single binary attribute to represent sex and gender of the transgender and gender nonbinary (TGNB) populations [6,7].

The inability to capture accurate sex and gender data in EHRs has also created a structural barrier to the health of the TGNB populations [2]. Without accurate sex and gender documentation, health care staff are unable to identify TGNB patients properly, which can lead to distress, stigma, and delay in seeking care for these patients [6,7]. The estimated TGNB populations in the United States range from 0.5% to 4.5% among adults and 2.5% to 8.4% among children and adolescents with upward trends noted, but the actual proportions can vary as many remain invisible in EHRs [8]. Jeffee et al [9] reported that 1 in 4 TGNB patients seeking health care services were denied equal treatment, and over 40% of transgender men experienced verbal harassment, physical assault, or denial of care. Studies have shown TGNB individuals to have worse health care access, quality, and outcomes, including lower rates of health maintenance screening and life expectancies, and higher rates of depression, substance use, and chronic illness [2,9,10]. For instance, in a study of 5135 US veterans diagnosed with gender dysphoria, the odds were 4 times greater for depression and 5 times greater for HIV seropositivity compared with controls [10]. The mismatch of one’s sex and gender can also be problematic with sex-based care guidelines such as pregnancy and prostate cancer screening tests. For transgender patients who have changed their recorded birth sex to match their current gender, the built-in EHR rules would often reject these tests for not having the expected sex value present [11-13].

There have been efforts to improve the definition, collection, and use of sex and gender data in EHRs among health care organizations. For instance, in 2013 the World Professional Association for Transgender Health published a set of recommendations for EHR developers, vendors, and users with respect to transgender patients [14]. In the United States, the Centers for Medicare and Medicaid Services and Office of the National Coordinator (ONC) for health information technology (IT) require EHR vendors to include sex and gender data fields as part of the EHR software certification [15], but health care providers are currently not required to collect this information [16]. Notable examples where gender identity is collected and used include the Fenway Institute [17] and the US Department of Veterans Affairs [11]. However, there are wide variations in current practices [8,18,19], and many health care organizations are yet to implement a system to collect sex and gender data [2]. The lack of common standards in defining sex and gender data fields and code values has also made it difficult to exchange and reuse this information across EHRs [20]. Overall, this is a complex multi-level challenge with numerous implementation barriers to explore for health policy, health systems, IT, providers, researchers, and patients.

### Objectives

The purpose of this environmental scan is to understand how sex and gender data are defined and implemented in existing Canadian EHR systems and current international health information standards. This study informs a larger initiative underway by the authors to modernize sex and gender information practices in Canadian EHRs. The need for this study is best summed up by the House of Commons Report on the Health of the Lesbian, Gay, Bisexual, Trans, Queer, Intersex, Asexual and Two-Spirit (LGBTQIA2+) communities, which states that “...the LGBTQIA2+ communities in Canada experience numerous health inequities...data collection [should] be improved in order to obtain a more complete picture of the health of gender and sexual minorities in Canada.” [21].

## Methods

### Definition of Terms

We conducted an environmental scan to examine public information sources on sex and gender definitions in Canadian EHRs and international health information standards. An environmental scan is a method of assessing the landscape on a specific topic based on multiple sources, including published literature, organizational documents, and key informants [22,23]. Sex and gender are broad terms used to include sex- and gender-related concepts such as sex at birth, gender identity, and pronouns [5,24]. Definitions refer to data element names, code systems, and value sets for these concepts. The data element name is the label for an attribute, code system is the scheme used to codify concepts, and the value set is a group of codes an attribute can hold [25,26]. EHRs refer to electronic collections of an individual’s lifetime health history and care records in the health ecosystem. They include input sources such as laboratory and pharmacy systems that collect individual health records, and administrative and clinical databases that house extracted records for health system analysis and reporting [27].

### Information Sources

This study was built on an earlier environmental scan report from the Canada Health Infoway [28]. For that report, Canada Health Infoway staff extracted sex and gender definitions in EHR-related data dictionaries from public websites of Canadian health jurisdictions and national health care organizations. They also received guidance from the Infoway Sex and Gender Working Group on additional EHRs to be included. The Working Group is a multi-sectoral virtual community representing government agencies, health care organizations, advocacy groups, care providers, private vendors, and academics across Canada interested in sex and gender issues in EHRs. Drawing on the Infoway report, we added sex and gender definitions from international standards communities involved with the development, implementation, or approval of health information standards.

### Data Analysis

For analysis, we summarized all sex and gender definitions from these sources and reviewed them for clarity, consistency, and accuracy. The review was conducted by the authors of this study with expertise or experience in patient care (KD), health information standards (FL), health equity (MA), and TGNB issues (RQ and KD). We also sought and received feedback on the definitions and earlier versions of this study from Infoway Sex and Gender Working Group members [12].

## Results

### Types of Information Sources

The information sources selected for this study consisted of public website descriptions of 52 databases and 55 data standards from 12 Canadian entities and 10 standards communities (Multimedia Appendix 1 [29-116]). The Canadian entities included (1) Alberta, British Columbia, Manitoba, Ontario, and Newfoundland and Labrador as 5 provincial jurisdictions responsible for 50 of these databases or data standards; (2) Canadian Institute for Health Information (CIHI) as the national health data holdings custodian responsible for 24 of these databases or standards; (3) Canadian Primary Care Sentinel Surveillance Network and Canadian Longitudinal Study on Aging as 2 national consortiums responsible for the database for more than 450 primary care practices and the database for long-term community health survey of more than 50,000 aging individuals, respectively; (4) Statistics Canada (StatCan) as the federal agency responsible for the national data standards for population surveys, census, and cancer registries; (5) Canada Health Infoway as the national organization responsible for Canadian health data exchange and terminology standards; and (6) Centre for Addictions and Mental Health (CAMH) and Tri-Hospital and Toronto Public Health (Tri-H+TPH) as 2 health care organizations considered leaders and early adopters of sex and gender documentation in EHRs.

The standards communities included (1) Health Level Seven (HL7), Diagnostic Imaging and Communication in Medicine (DICOM), and the International Organization for Standardization as 3 international standards organizations that develop data exchange standards including HL7 Version 2 (V2) and Version 3 (V3), Clinical Document Architecture, Fast Healthcare Interoperable Resources (FHIR), and DICOM medical imaging standards; (2) Systemized Nomenclature of Medicine (SNOMED) International and Logical Observations Investigations Names and Codes (LOINC) as 2 international organizations that develop health terminology standards; (3) OpenEHR as an international collaboration that has developed the Gender Archetype, and the BioPortal where the Gender, Sex, and Sexual Orientation (GSSO) ontology developed by Kronk [111] is hosted; (4) ONC, National Health Services, and Australian Institute of Health and Welfare as 3 government agencies considered leaders in developing health information standards.

### Sex and Gender Definitions in Existing Canadian EHRs

#### Sex

We extracted sex definitions and summarized them by data element, code system, and value set (Multimedia Appendix 2). There are 25 entries with 7 unique data element names. After merging names with minor variations, 5 distinct data element names remained. After eliminating duplicate entries, 7 code systems and 21 unique value sets remained (Textbox 1). Most variations are when sex is unknown, undifferentiated, other, or has no information. For instance, unknown is coded as *U*, *UNK*, *3*, or *39*; undifferentiated is coded as *UN*, *U*, or *1*. The same code can also have different values. For example, *U* can be *Unknown*, *Unknown or Undifferentiated*, *Undifferentiated*, or *Undetermined*; *UN* can be *Unknown*, *Undifferentiated*, or *Not assigned male or female*. Other can be *Other*, *Other (including hermaphrodites* and *transsexual)*, or *Other (person could not be uniquely identified)*. When no information is given, different codes and values are used, including *9-Not stated*, *281-Null*, and *blank*. From the definitions, it is unclear if *Undifferentiated*, *Undetermined*, and *Indeterminate* refer to the same concept.

**Summary of definitions for sex in Canadian electronic health records.**

**Data element name (n=5)**

- Sex
- Administrative Sex
- Patient’s sex
- Sex assigned at Birth
- Infant’s sex

**Code system (n=7)**

- Health level Seven version 2 (HL7V2)-0001
- Diagnostic imaging and communication in medicine (0010,0040)
- Canadian Institute for Health Information
- Statistics Canada
- Newfoundland & Labrador Centre for Health Information
- Canadian Primary Care Sentinel Surveillance Network
- Manitoba

**Value set (n=21)**

- Male, female
- M-male, F-female
- M-male, F-female, blank (if unknown)
- M-male, F-female, I-indeterminate, UNK-unknown
- M-male, F-female, O-other
- M-male, F-female, O-other (trans-sexual, hermaphrodite)
- M-Male, F-Female, O-Other (person could not be uniquely identified as male or female; eg, hermaphrodite)
- M-male, F-female, U-unknown
- M-male, F-female, U-unknown, O-other
- M-male, F-female, U-unknown/undifferentiated, O-other
- M-male, F-female, U-undetermined, unknown or other
- M-male, F-female, U-undifferentiated, 9-not stated
- M-male, F-female, UN-not assigned male or female
- M-male, F-female, UN-undifferentiated, UNK-unknown
- 1-male, 2-female
- 1-male, 2-female, 3-other
- 1-male, 2-female, 3-unknown
- 1-male, 2-female, O-other
- 1-male, 2-female, O-other (includes hermaphrodites, transsexual)
- 1-male, 2-female, U-unknown
- 155,939-female, 133,338-male, 281-null, 39-unknown, 1-undifferentiated

#### Gender

We extracted gender definitions and summarized them by data element, code system, and value set (Multimedia Appendix 3). There are 35 entries with 12 unique data element names. After merging names with minor variations, 5 distinct names remained. After eliminating duplicate entries, 9 code systems and 22 unique value sets remained (Textbox 2).

**Summary of definitions for gender in Canadian electronic health records.**

**Data element name (n=5)**

- Gender
- Administrative Gender
- Clinical Gender
- Gender identity
- Newborn gender

**Code system (n=9)**

- Health Level Seven version 2
- Health Level Seven version 3
- Clinical Document Architecture
- Fast Healthcare Interoperable Resources
- Canadian Institute for Health Information
- Alberta
- Manitoba
- Newfoundland & Labrador
- Canadian Longitudinal Study on Aging

**Value set (n=22)**

- M-male, F-female
- M-male, F-female, D-gender diverse, UNK-not known, NA-not applicable
- M-male, F-female, I-undifferentiated stillbirth only, U-unknown, O-other; for trans-sexual or hermaphrodite, U-undifferentiated; for stillbirths only (discontinued)
- M-male, F-female, O-other (trans-sexual or hermaphrodites)
- M-male, F-female, OTH-other gender identity, UNK-not known, NA-not applicable
- M-male, F-female, U-unknown
- M-male, F-female, U-unknown, O-other
- M-male, F-female, U-unknown, O-other, I-indeterminate
- M-male, F-female, U-undifferentiated
- M-male, F-female, U-undifferentiated stillbirth only, O-other (trans-sexual or hermaphrodite)
- M-male, F-female, U-undifferentiated, stillbirths only, O-other or unknown
- M-male, F-female, UN-undifferentiated
- M-male, F-female, UN-undifferentiated, UNK-unknown
- M-male, F-female, UN-undifferentiated, UNK-unknown, NI-no information (could not be uniquely defined as male or female, eg, hermaphrodite), OTH-other
- M-male, F-female, 7-not collected, 9-unknown
- Male-male, Female-female, Unknown-unknown
- Male-male, Female-female, Unknown-unknown, Other-other
- Male-male, Female-female, refused or something else
- Male-male, Female-female, Other-other, Unknown-unknown
- 1-male, 2-female, 3-unknown
- 1-male, 2-female, 8-don’t know/no answer, 9-refused
- 1-male, 2-female, ZZ-other

Most variations are in undifferentiated, unknown, other, and no information is given. For instance, undifferentiated is coded as *UN* or *U*; unknown as *Unknown*, *U*, *UNK*, *3*, or *9*; and other as *O*, *OTH*, *Other*, or *ZZ.* There are also different values for undifferentiated and other, regardless of whether the same code is used or not. For instance, the value for undifferentiated can be *Undifferentiated*, *Undifferentiated still birth only*, or *Undifferentiated (could not be uniquely defined as male or female, eg, hermaphrodite)*. Other can be *Other*, *Other* *or unknown*, *Other (trans-sexual or hermaphrodites)*, or *Other gender identity*. When no information is given, different codes and values are used, including *7-Not collected*, *8-Don’t know or no answer*, *9-Refused*, *Refused*, or *something else*, *NA-Not applicable*, or *NI-No information*. From the definitions, it is unclear if *Undifferentiated* and *Indeterminate* refer to the same concept.

### Expanded Sex and Gender Definitions in Canadian Health Care Organizations

We extracted expanded sex and gender definitions in policy and practice guides of 4 Canadian institutions (Multimedia Appendix 4) and summarized them by organization, concept, and value set (Table 1).

**Table 1:** Expanded sex and gender definitions in Canadian health care organizations.

Concept (n=10)		Organization (n=4)	Value set and/or description if available (n=10)
**Sex**			
	Sex	StatCan^a^	M-male, F-female
	Variant of sex	StatCan	M-male, F-female, I-intersex
	Sex at birth	CIHI^b^	M-male, F-female, I-indeterminate, UNK-unknown
	Intersex	CIHI	Refers to a variety of conditions where a person has atypical development of sex characteristics, such as reproductive anatomy, sex chromosomes or sex-related hormones, that is not consistent with typical definitions of male of female
**Gender**			
		StatCan	M-male gender, F-female gender, D-gender diverse
		CIHI	M-male, F-female, D-gender diverse, UNK-unknown, NA-not applicable
		Tri_Hospital + TPH^c^	Male, female, trans-female to male, trans-male to female, intersex, other (specify), prefer not to answer, do not know
**Cisgender**			
		StatCan	C-cisgender, CM-cisgender man, CF-cisgender woman
**Transgender**			
		StatCan	T-transgender, TM-transgender man, TF-transgender woman, TG-transgender person, non-exclusive category
**Nonbinary gender**			
		StatCan	Transmasculine demi-boy, transfeminine demi-girl, pan-gender poly-gender bi-gender two-spirit, gender-fluid neutrois, genderless, agender
**Lived gender**			
		CIHI	How a person publicly presents their gender, which include behavior, appearances, name and pronoun
**Gender identity**			
		CIHI	A person’s internal and experience, a sense of being a woman, man, both, neither or along a spectrum
		CAMH^d^	Male, female, transsexual, transgender, genderqueer, two-spirit, FTM (female-to-male), MTF (male-to-female), intersex, unsure, questioning, other, prefer not to answer

^a^StatCan: Statistics Canada.

^b^CIHI: Canadian Institute for Health Information.

^c^TPH: Toronto Public Health.

^d^CAMH: Centre for Addiction and Mental Health.

For sex, StatCan and CIHI have identified intersex as a distinct concept. For value sets, StatCan has defined *I-Intersex* as one of the sex options, whereas CIHI has *I-Indeterminate* as one of the sex at birth options. For gender, both StatCan and CIHI have *D-Gender Diverse* as a distinct concept. StatCan further distinguished gender into categories of cisgender, transgender, and nonbinary gender with specific value sets such as *CM-Cisgender man*, *TM-Transgender man* and *Trans-Masculine*, and *Demi Boy*. CIHI has distinguished between gender identity and lived gender but only defined a value set for gender with *D-Gender Diverse* as an option. For CAMH and Tri-H+TPH, their gender value sets have many options but with some variations: only CAMH has options for *Transsexual*, *Transgender*, *Genderqueer*, and *Questioning.* There are also variations when a person’s sex or gender is not recorded: *UNK-Unknown*, *NA-Not Applicable*, *Other*, *Prefer not to answer*, or *Do not know.*

### Sex and Gender Definitions in Standards Communities

#### Definitions

We extracted sex and gender definitions published by 10 standards communities (Multimedia Appendices 5 and 6). The published standards ranged from those in routine use such as HL7V2, V3, and DICOM specifications, to recently proposed specifications from the FHIR, HL7 Gender Harmony Project, ONC-Interoperability Standards Advisory, OpenEHR Gender Archetype, and BioPortal-GSSO. For sex, there are 11 unique data element names from 13 code systems and 16 value sets (Textbox 3). GSSO has also defined 3 sex subtypes as biological sex, anatomic sex, and genotypic sex. For gender, there are 10 unique data element names from 11 code systems and 12 value sets (Textbox 4).

**Summary of definitions for sex-related concepts from standards communities.**

**Data element names (n=11)**

- Sex
- Administrative sex
- Patient’s sex
- Sex of patients
- Patient sex (at birth)
- US-core-birthsex
- Sex assigned at birth
- Sex for clinical use
- Biological sex
- Anatomic sex
- Genotypic sex

**Code systems (n=13)**

- International Organization for Standardization-5218
- Australian Institute of Health and Welfare–Metadata Online Repository
- Diagnostic Imaging & Communication in Medicine (DICOM)–Context Identifier 7455
- DICOM- (0010,0040)
- DICOM- proposed
- BioPortal- gender sex and sexual orientation ontology
- Health Level 7 (HL7)V2-0001
- HL7V3
- Fast Healthcare Interoperability Resources
- National Health Services
- Logical Observations Investigations Names and Codes
- OpenEHR
- HL7 Gender Harmony

**Value sets or subtypes (n=16)**

- 1-male, 2-female, 0-not known, 9-not applicable
- 1-male, 2-female, 3-other, 9-not stated or inadequately described; alternate scheme
- M-male, F-female, X-other
- M-male, F-female, U-unknown sex, MP-male pseudohermaphrodite, FP-female pseudohermaprodite, H-hermaphrodite, MC-male changed to female, FC-female changed to male, 121104-ambiguous sex, 12102-other sex, 121103-undetermined sex
- M-male, F-female, A-ambiguous, N-not applicable, O-other, U-unknown
- M-male, F-female, O-other
- M-male, F-female, O-nonbinary, eg, intersex, other situations where neither male nor female apply clinically
- 1-male, 2-female, 8-not specified, 9-home leave (forward operational plans only)
- 76689-9 Sex assigned at birth
- M-male, F-female, UNK-unknown
- Male, female, intersex
- Male, female, nonbinary, unknown
- Sex subtypes (biological sex, anatomic sex, genotypic sex)
- Biological sex subtypes (chromosomal sex, anatomic sex, sex for imaging, hormonal or organ status sex)
- Anatomic sex subtypes (gonadal sex)
- Genotypic sex subtypes (chromosomal sex, genetic sex)

**Summary of definitions for gender-related concepts from standards communities.**

**Data element names (n=10)**

- Gender
- Gender identity
- Administrative gender
- Person stated gender
- Code recorded gender or sex identity (previously legal gender)
- Legal gender
- Gender expression
- Affirmed gender
- Assigned gender
- Preferred pronoun

**Code systems (n=11)**

- Health Level 7 V3
- Fast Healthcare Interoperability Resources
- Diagnostic Imaging & Communication in Medicine
- Metadata Online Repository
- OpenEHR
- ISO-5218
- National Health Services
- Logical Observations Investigations Names and Codes
- Systemized Nomenclature of Medicine Clinical Terms
- Gender Sex and Sexual Orientation Ontology
- Office of National Coordinator Interoperability Standards Advisory

**Value sets (n=12)**

- M-male, F-female, UN-undifferentiated
- Male-male, Female-female, Other-other, Unknown-unknown
- 1-male, 2-female, 9-indeterminate (unable to be classified as either male or female), X-not known (not recorded)
- Man, woman, boy, girl, non-binary, notExpressed (examples only)
- 1-male (including trans man), 2-female (including trans woman), 3-non-binary, 4-other (not listed), Z-not stated (person asked but declined to provide a response)
- Transgender-female, transgender-male, non-binary, male, female, other, non-disclose
- 77691-5 gender identity
- 446151000124109|Male gender|, 446141000124107| Female gender|, 407377005|Femalte-to-male (FTM)/Transgender male/Trans man, 407376001|Male-to-female (MTF)/Transgender female/Trans woman|, 446131000124102| Identifies as non-confirming gender|, (US synonyms Include Genderqueer, Identifies as neither exclusively male nor female, Non-binary gender), OTH-Additional gender category or other, please specify, ASKU-Choose not to disclose
- Affirmed gender, assigned gender, assumed gender, authentic gender, felt gender, legal gender, natal gender
- Affirmed female, affirmed male
- Assigned female at birth, assigned male at birth
- Pronoun examples only—she, he, they, ze

#### Value Sets

There are variations in sex and gender value options. Examples are *M-Male* and *1-Male* for male, and *UN-Undifferentiated*, *9-Indeterminate*, *X-Other*, *X-Not known (not recorded)*, *0-Not known*, or *O-Non binary* if no information is available. When compared with more established standards such as HL7 V2 and DICOM, those from ONC, OpenEHR, and GSSO have more sex and gender value options. For instance, ONC has defined gender identity and sex assigned at birth value sets from LOINC and SNOMED CT. Examples include LOINC code *77691-5* for *Gender identity* and SNOMED CT code *407377005|Female-to-male transsexual|*. GSSO has more sex-related value sets based on biological features such as anatomical, chromosomal, brain, and genetic sex.

## Discussion

### Principal Findings

#### Overall Issues

There are wide variations in the definition and implementation of sex and gender in Canadian EHRs and international health information standards. There is a lack of clarity in some sex and gender concepts. There is inconsistency in the data element names, code systems, and value sets used to represent sex and gender concepts across EHRs. The appropriateness and adequacy of some value set options are questioned as our societal understanding of sexual health continues to evolve. Outdated value set options raise concerns about current EHRs supporting the provision of culturally competent, safe, and affirmative health care. The limited options available also perpetuate the inequities faced by the TGNB populations. The expanded sex and gender definitions from leading Canadian organizations and international standards communities have also brought implementation challenges in migrating these definitions into existing EHRs. These findings are elaborated below.

#### Lacking Conceptual Clarity

A major issue is the conflated use of sex and gender as a discrete single binary concept to define sex and gender identity. This longstanding practice can create confusion when a person’s biology of being male or female along a spectrum is different from one’s identity or feeling of one’s gender identity as a woman, man, neither, both, or something else. The use of clinical gender, administrative gender, and administrative sex to represent sex and gender concepts for specific use is problematic because their context for use is often not well defined. As an example, the historical reliance on administrative gender as a means to assign hospital beds is inappropriate for transgender persons. The expanded sex and gender concepts from standard communities such as intersex, genotypic sex, and gender identity (eg, neutrois) are unfamiliar and sometimes overlapping terms that can be confusing to most but some clinicians and staff with specialty knowledge in sex and gender issues. These findings are consistent with the review by Jeffee [9], where care providers lacked familiarity with the terms and were ill-prepared to collect sex and gender data. They also echo the need for “accurate use of [sex- and gender-based medicine] terminology in research and clinical practice across medical specialties and across health professions” [5].

#### Inconsistent Definitions

Similar to what is reported in the literature [3,117], there is a great deal of semantic variability in the data element names, code systems, and value sets used to represent sex and gender concepts in EHRs. For instance, sex, sex code, administrative sex, and patient’s sex may all refer to a person’s biology along a spectrum. However, different value set options exist across systems, including *indeterminate*, *intersex*, *undifferentiated*, and *not assigned male or female when the person is not male or female*. For gender, it is unclear if *undifferentiated*, *undifferentiated stillbirth only*, and *indeterminate* are equivalent terms. Even when sex and gender information is not available, it is unclear if such options as *unknown*, *no information*, *not collected*, *not applicable*, or *<blank>* have the same meaning. Although some value sets are from the same code systems such as DICOM and HL7, they can contain different options with little explanation (see Textbox 3). The HL7 Gender Harmony Project also noted these discrepancies in their recent work to standardize sex and gender definitions [118].

#### Inappropriate Value Options

As our understanding of sex and gender evolves over time, some historic value options are now considered offensive or outdated [119,120]. Terms such as *hermaphrodite* and *natal* are now recognized as being offensive. *M2F* and *F2M* are regarded as outdated by some and should be replaced by *transfeminine* and *transmasculine*. Similarly, *intersex* and *indeterminate* should not be interchangeable terms. Misuse of the label preferred pronouns over pronouns as a data element name is also common [9,120].

#### Evolving Value Options

As our society gives greater attention to sex and gender identity, the range of value options for sex and gender continues to expand. Dunne et al [7] reported that care providers and patients cited the need for a broader range of gender options and birth-assigned sex in EHRs. For use contexts, such terms as cisgender, transgender, and gender diversity may be adequate for classifying and reporting gender profiles at the population level. However, when caring for individual patients, distinguishing specific gender identity can be central to understanding health status, planning appropriate care, and maintaining patient safety. The proposed use of men and women as gender options instead of males and females also requires major shifts in society’s discourse on sex and gender labeling [5,9]. As we broaden the range of value options available, the notion of third gender in other cultures such as Hijra in India also needs to be considered. To support culturally competent, equitable care requires expanding sex and gender options as an ongoing practice [7,121].

#### Implementation Challenges

There is no mandate in Canada on the type of sex and gender data to be collected in EHRs at present. Introducing expanded sex and gender definitions into existing EHRs can be a major undertaking for health care organizations, given the competing demands, limited resources, and redevelopment effort required. Implementation efforts to date suggest that sex and gender concepts are foundational in nature, and implicate a myriad of technical, organizational, and societal aspects [17,122]. Technically, there is the potential for modernized definitions to affect all EHRs. As such, consensus on relevant sex and gender definitions is needed, and the implementation effort should be well coordinated. There needs to be a trusted source where one can validate the origin and provenance of the recorded sex and gender data in different EHRs to ensure their accuracy. Organizationally, the implementation plan and rationale for change must be clearly articulated to care providers, support staff, patients, and others affected. Education and training on the rationale, policies, and practices involved in collecting and using this information are necessary to cultivate a culturally safe and equitable environment and to prevent potential harm such as misgendering, outing, and deadnaming [5-7]. At the societal level, we need to engage the public to raise their awareness of this change and explain how it may affect their day-to-day interactions with the health ecosystem [9,15].

### Proposed Actions

#### Need for Actions

On the basis of these findings, we propose a set of high-level actions to modernize sex and gender definitions in Canadian EHRs. These proposed actions are meant to initiate a dialogue with stakeholders, including individuals, communities, and organizations involved with developing or implementing, or affected by, the definitions. Collectively, they represent a set of guiding principles for organizations wishing to strive toward an inclusive EHR. These actions are also consistent with those reported by early adopters and leading organizations, including Fenway Health [17], Veteran Health Administration [11], University of California Davis Health System [122], and Canada’s St. Michael’s Hospital [123], that have started collecting expanded sex and gender data in their EHRs. Our proposed actions are described below.

#### Articulate the Need

Articulate the need for this work by explaining why the proposed actions are necessary to improve the health of sexual and gender minorities in Canada. These include the rationale for the collection of modernized sex and gender information in EHRs, how this information can advance our understanding of this population, and provide safe and culturally competent care. For instance, having expanded sex and gender definitions in the EHR is seen as an organization’s obligation for inclusion as part of the Canadian Human Rights Code and Charter of Rights and Freedom [124].

#### Reach Consensus on Relevant Concepts

Reach consensus on relevant sex and gender concepts that can improve the health of Canadians, especially the underserved sex and gender minority populations. These concepts should address the need from policy, practice, research, and community perspectives [5]. Guidance from clinicians who use the data at the patient care level will be critical. This will help determine what codes and values are necessary versus what changes could occur at the policy level. Key concepts to consider include sex, sex assigned at birth, gender, administrative gender, gender identity, pronouns, and such variants as current sex or gender, gender expression, and legal sex or gender.

#### Reach Consensus on Expanded Definitions in EHRs

Reach consensus on expanded sex and gender definitions in EHRs with respect to data element names, code systems, and value sets [14,26,27]. For instance, the use of subtypes and synonyms for data element names may improve shared meaning while allowing flexibility in local systems. Further harmonization of code systems and value sets to represent sex and gender concepts may facilitate wider adoption. An example is the development of reference sex-gender code systems and value sets with cross-maps to those used in local systems to guide the transition. Another is to allow write-in options at the patient care level that can be rolled-up for analysis and reporting without suppressing important data.

#### Develop a Coordinated Plan

Develop a coordinated action plan to implement expanded sex and gender definitions in Canadian EHRs. This plan should be action-oriented, stakeholder- and consensus-driven, and achievable in stages over time, given the limited resources and competing priorities in EHR redevelopment work [17,20]. The plan should also be coordinated with nonhealth government agencies where sex and gender data are collected (eg, driver’s licenses and passports). Examples include expanding or harmonizing sex-gender concepts, creating reference sex-gender definitions with cross-maps to local systems, conducting small-scale implementation with selected EHRs, collaborating with standards communities, and sharing the lessons.

#### Embrace EHR Change From Socio-Organizational-Technical Aspects

Embrace EHR changes from socio-organizational and technical aspects to ensure success. Modernizing sex and gender definitions in EHRs is more than just a technical endeavor on data standardization but one that involves complex interplay of various social and organizational dynamics [125]. Organizations embarking on this journey need to engage senior executives, clinical leaders, and TGNB communities to cocreate an inclusive environment with culturally competent staff who are respectful of diversity, familiar with TGNB terminology, and knowledgeable in unique care needs of TGNB patients [126]. Key success factors include having explicit policy and practice guidelines on inclusion, ongoing staff education and training, a flexible EHR, and patient and public awareness to address the transition [11,15].

#### Demonstrate Benefits

Demonstrate the benefits in tangible terms by establishing relevant and meaningful measures to evaluate the impact of these expanded definitions in EHRs on individuals, communities, organizations, and the health ecosystem over time. The measures should be multifaceted covering policy, practice, research, and community perspectives. Knowledge translation is an essential element of this evaluation process to share the results and lessons learned. Examples of measures include adherence to best practices and respecting protected human rights, data completeness in EHRs, perceived usefulness by patients and providers, and quantified improvements in health risks, service use, and health status.

### Study Limitations

This study has some limitations. First, the information sources were restricted to public documents on existing EHRs and standards. To keep the study manageable, we excluded systems such as local EHRs, federal EHRs (eg, correctional facilities), and citizens’ registries. Second, our study focused only on documented sex and gender definitions but not the process or context for their collection and use. The depth of our analysis is limited as there are deep societal, technical, ethical, and legal aspects that require more in-depth and thoughtful deliberations. Third, the environmental scan was based on the earlier Infoway report and key informant input. The work and results are not reproducible by others. Fourth, although we received valuable feedback from the virtual community, the study could benefit from wider consultations with other stakeholders concerned with sex and gender representation in EHRs. Finally, our proposed actions are presented only at a high level based on the findings of this scan. Sex and gender are complex, with implications for culture, technology, and society. Much work still lies ahead to design EHRs in ways that can support health equity, especially for the TGNB populations.

### Conclusions

In this study, we examined sex and gender in Canadian EHRs and international health information standards. There are wide variations in how these concepts are represented and defined. The case to modernize sex and gender definitions in EHRs is compelling. Respecting the human rights aspect as well as safe, accurate, and efficient use, exchange, and reuse of sex and gender information for all populations is one condition for health equity that can be improved in a very direct way. A coordinated stakeholder- and consensus-driven action plan is needed for this effort to help advance the health of Canadians, especially the TGNB populations.

## Appendix

Multimedia Appendix 1 List of information sources.

Multimedia Appendix 2 Existing sex definitions in Canadian electronic health records.

Multimedia Appendix 3 Existing gender definitions in Canadian electronic health records.

Multimedia Appendix 4 Expanded sex and gender definitions in Canadian health care organizations.

Multimedia Appendix 5 Published definitions for sex-related concepts from standards communities.

Multimedia Appendix 6 Published definitions for gender-related concepts from standards communities.

## Abbreviations

CAMH: Centre for Addictions and Mental Health
CIHI: Canadian Institute for Health Information
CIHR: Canadian Institutes of Health Research
CT: clinical terms
DICOM: diagnostic imaging and communications in medicine
EHR: electronic health record
FHIR: fast healthcare interoperability resources
GSSO: gender, sex, and sexual orientation
HL7: health level seven
IT: information technology
LGBTQIA2+: lesbian, gay, bisexual, transgender, queer or questioning, intersex, asexual, two-spirit
LOINC: logical observation identifiers, names, and codes
ONC: Office of the National Coordinator
SNOMED: systemized nomenclature of medicine
StatCan: Statistics Canada
TGNB: transgender and nonbinary
TRI-H+TPH: Tri-Hospital and Toronto Public Health
