# Correction: An Environmental Scan of Sex and Gender in Electronic Health Records: Analysis of Public Information Sources

**DOI:** 10.2196/30764

**Published:** 2021-06-04

**Authors:** Francis Lau, Marcy Antonio, Kelly Davison, Roz Queen, Katie Bryski

**Affiliations:** 1 School of Health Information Science University of Victoria Victoria, BC Canada; 2 Canada Health Infoway Toronto, ON Canada

In “An Environmental Scan of Sex and Gender in Electronic Health Records: Analysis of Public Information Sources” (J Med Internet Res 2020;22(11):e20050) the authors noted three errors.

1. Under the “Results” section, second paragraph of “Types of Information Sources” subsection, the statement *“(3) OpenEHR and BioPortal as 2 international collaborations that have developed the Gender Archetype and Gender, Sex, and Sexual Orientation (GSSO) ontology, respectively;”* was published incorrectly. This has now been corrected as follows:

(3) OpenEHR as an international collaboration that has developed the Gender Archetype, and the BioPortal where the Gender, Sex, and Sexual Orientation (GSSO) ontology developed by Kronk [111] is hosted;

2. Reference 111 was originally published as follows:

Gender, Sex and Sexual Orientation Ontology – Gender and Sex. BioPortal. URL: https://bioportal.bioontology.org/ontologies/GSSO/?p=classes&conceptid=root [accessed 2020-10-27]

This has been corrected to:

Kronk CA, Dexheimer JW. Development of the Gender, Sex, and Sexual Orientation ontology: Evaluation and workflow. J Am Med Inform Assoc 2020 Jul 01;27(7):1110-1115. [doi: 10.1093/jamia/ocaa061] [Medline: 32548638]

3. In the original article, the phone number of the corresponding author was published with a duplicate country code as *'1 12504725131'*.

This has been corrected to *'1 2504725131'*.

The correction will appear in the online version of the paper on the JMIR Publications website on June 4, 2021, together with the publication of this correction notice. Because this was made after submission to PubMed, PubMed Central, and other full-text repositories, the corrected article has also been resubmitted to those repositories.

