# Supplementary material for: An Environmental Scan of Sex and Gender in Electronic Health Records: Analysis of Public Information Sources
Source: J Med Internet Res. 2020 Nov 11;22(11):e20050. doi: 10.2196/20050 (PMC7688387; doi:10.2196/20050)
Supplement: Multimedia Appendix 1 [file jmir_v22i11e20050_app1.docx]

**APPENDICES – Online Supplementary Files.**

Appendix 1 - List of Information Sources

Appendix 2 - Existing sex definitions in Canadian EHRs

Appendix 3 - Existing gender definitions in Canadian EHRs

Appendix 4 - Expanded sex and gender definitions in Canadian health organizations

Appendix 5 - Published definitions for sex-related concepts from standards communities

Appendix 6 - Published definitions for gender-related concepts from standards communities

Appendix 1 - List of Information Sources.

1. EHRs and Standards – Jurisdictions in Canada

| Jurisdiction | EHR system and Standard | Source | Type |
| --- | --- | --- | --- |
| Alberta | Health Information Standards Committee for Alberta (HISCA) Standards (list of standards)  National Ambulatory Care Reporting System and Comprehensive Ambulatory Care Classification System Data Set Version 1.0, 2012  Chronic Disease Management Data Standards, Version 7.4 Appendix Set, Appendix E Code Tables V7.6, 2006  Continuing Care Data Standard Version 1.0, 2013  Stakeholder Client Data Set Version 1.4.2, 2013  Stakeholder Demographic Data Standard Version 2.2, 2003  Stakeholder Provider Data Set Version 1.5, 2018  Alberta Diagnostic Imaging Message Specification, Version 0.1, 2008  Alberta Diagnostic Imaging Reporting Requirement Data Standard, Version 0.1, 2008  Medical Summary for Transfer of Patient Data Version 0.1, 2005  Emergency Health Services Patient Care Reporting Minimum Data Set Version 1.2, 2011  Adverse Events Following Immunization Data Set Version 2.2, 2019  Breast Cancer Screening Minimum Data Set version 0.8, 2000  Communicable Disease and Outbreak Data Set, Draft, Version 1.0, 2014  Generic Screening Minimum Data Set Version 0.4, 2000  Immunization Data Set Version 1.6, 2019  Newborn Metabolic Screening Data Set Version 3.5, 2018  Perinatal Health Program Data Set Version 1.1, 2009 | Aa01  Aa02  Aa03  Aa04  Aa05  Aa06  Aa07  Aa08  Aa09  Aa10  Aa11  Aa12  Aa13  Aa14  Aa15  Aa16  Aa17  Aa18 | D  S  S  D  S  D  S  S  S  D  D  D  D  D  D  D  D |
| British Columbia | Client Registry Conformance Standards, Vocabulary HL7 Terminology Worksheet, R02.04.02, 20010  Provider Registry Conformance Standards, Vocabulary HL7 Terminology Worksheet, R02.04.01, 2009  Provincial Laboratory Info System Conformance Standards, Vocabulary HL7 Terminology, V01R03, 2008  EMR to EMR Data Transfer & Conversion Standard, Part II Consolidated CDA Implementation Guide V1.35, 2013  Conformance Standards PharmaNet v03 Terminology Worksheet, 2019 | Aa19  Aa20  Aa21  Aa22  Aa23 | S  S  S  S  S |
| Manitoba | EMR Certification Baseline Requirements Specification, Version 1.9, 2019  eChart Manitoba Context-Sensitive Launch Interface Requirements Specification Version 1.1, 2016  EHealth_hub Laboratory Result Distribution Interface Requirements Specification Version 1.3, 2016  EHealth_hub Diagnostic Imaging Result Distribution Interface Requirements Specification Version 1.3, 2016  eHealth_hub Client Registry Query Service Interface Specification Version 1.1, 2019  Primary Care Quality Indicator Reminders and Data Extract Specification Version 1.6, 2018  Interprofessional Team Data Extract Specification Version 1.0, 2015 | Aa24  Aa25  Aa26  Aa27  Aa28  Aa29  Aa30 | S  D  D  D  D  S  S |
| Newfoundland & Labrador | Provincial Discharge Abstract Database User Guide v1.0, 2017  Live Birth System 2016 Master Methodology v1.0, 2017, Appendix 1 – NLCHI Live Birth System Data Dictionary  Mortality System Data User Guide v1.0, 2017, Appendix 1 – NLCHI Mortality System Data Dictionary  Stillbirth System 2016 User Guide v1.0, 2017, Appendix 1 – Stillbirth System Data Dictionary | Aa31  Aa32  Aa33  Aa34 | D  D  D  D |
| Ontario | Acute and Community Clinical Data Repository (acCDR) Input Standard Version 2.5.1, 2018  Ontario Oncology Information System Standards: Defining its Meaningful Use, Mar 31, 2014  Diagnostic Imaging Common Service Standard, Release 2 CDA & XDS Implementation Guide Version 1.2, 2014  Ontario Laboratories Information System (OLIS) Standard – OLIS Interface Specification, Version R01.24, 2015  Provincial Client Registry (PCR) Standards Release 2 – PCR ADT HL7V2 Update, Version 10.9, 2016 (v2)  Provincial Client Registry (PCR) Standards Release 2 – PCR Implementation Guide, Version 1.2, 2013 (v3)  Provincial Clinical Documentation Architecture (CDA) Header Standard –Implementation Guide, V1.0, 2014  Provincial Provider Registry Specification, HL7V3 Implementation Guide & Terminology Worksheet, V1.0, 2016-7  Digital Health Drug Repository (DHDR) Specification FHIR –DHDR Release 2 HL7 FHIR Implementation Guide & Terminology Worksheet, V2.0.0, 2017  DHDR Specification FHIR –Point of Care Systems Access HL7 FHIR Implementation Guide, Version 3.0.1, 2019  Digital Health Immunization Repository (DHIR) Consumer Access HL7 FHIR Implementation Guide V4.0.1, 2019  DHIR Specification FHIR (Release 1) - HL7 FHIR Implementation Guide Version 3.0.4, 2017  DHIR Specification FHIR (Release 4) – Point of Care System Access FHIR Implementation Guide V4.0.1, 2019  Provincial Client Registry (PCR) HL7 FHIR Implementation Guide - v1.0.0  Provincial Provider Registry (PPR) –FHIR – PPR HL7 FHIR Implementation Guide v1.0.0 draft  OntarioMD EMR Specifications – EMR Core Data Set Version 5.0, Logical Data Dictionary 2017  OntarioMD Electronic Medical Record Hospital Report Manager 4.3 Requirements, Version 1.0, 2017 (EMR HRM) | Aa35  Aa36  Aa37  Aa38  Aa39  Aa39  Aa40  Aa41  Aa42  Aa43  Aa44  Aa45  Aa46  Aa47 Aa48  Aa49  Aa50 | D  D  D  D  D  S  S  D  S  D  D  S  S  S  S  D  S |

Legends: S-Standard specifications, D-Database repository

Reference Sources. EHRs and Standards – Jurisdictions in Canada

Aa01: Alberta Government. Health Information Standards Committee - HISCA Standards. Available from <https://www.alberta.ca/hisca-standards.aspx>

Aa02: Alberta Government. NACRS and CACS data standards. Version 1.0. Available from <https://open.alberta.ca/publications/nacrs-and-cacs-data-standard-version-1>

Aa03: Alberta Government. Chronic disease management data standards. Version 7.3. Version 1.0. Available from <https://open.alberta.ca/publications/chronic-disease-management-data-standard-version-7-3>

Aa04: Alberta Government. Alberta Continuing Care Information System data standard. Version 1.0. Available from <https://open.alberta.ca/publications/alberta-continuing-care-information-system-data-standard-version-1-0>

Aa05: Alberta Government. Stakeholder client data standard. Version 1.4.2. Available from <https://open.alberta.ca/publications/stakeholder-client-data-standard-version-1-4-2>

Aa06: Alberta Government. Stakeholder demographic data standard. Version 2.2. Available from <https://open.alberta.ca/publications/stakeholder-demographic-data-standard-version-2-2>

Aa07: Alberta Government. Stakeholder provider data standard. Version 1.5. Available from <https://open.alberta.ca/publications/stakeholder-provider-data-standard-version-1-5>

Aa08: Alberta Government. Diagnostic imaging HL7 message specifications. Version 0.1. Available from <https://open.alberta.ca/publications/diagnostic-imaging-hl7-message-specifications-version-0-1>

Aa09: Alberta Government. Alberta diagnostic imaging reporting requirement data standard. Version 0.1. Available from <https://open.alberta.ca/publications/alberta-diagnostic-imaging-reporting-requirement-data-standard-version-0-1>

Aa10: Alberta Government. Medical summary for transfer of patient data. Version 0.1. Available from <https://open.alberta.ca/publications/medical-summary-for-transfer-of-patient-data-version-0-1>

Aa11: Alberta Government. Emergency health services: patient care reporting minimum data standard. Version 1.2. Available from <https://open.alberta.ca/publications/emergency-health-services-patient-care-reporting-minimum-data-standard>

Aa12: Alberta Government. Adverse Events Following Immunization data standard [Version 2.2]. Available from <https://open.alberta.ca/publications/adverse-events-following-immunization-data-standard-version-2-2>

Aa13: Alberta Government. Breast cancer screening minimum data set. Version 0.8. Available from <https://open.alberta.ca/publications/breast-cancer-screening-minimum-data-set-version-0-8>

Aa14: Alberta Government. Communicable disease and outbreak data standard. Version 1.0. Available from <https://open.alberta.ca/publications/communicable-disease-and-outbreak-data-standard-v1-0>

Aa15: Alberta Government. Generic screening minimum data standard. Version 0.4. Available from <https://open.alberta.ca/publications/generic-screening-minimum-data-standard-version-0-4>

Aa16: Alberta Government. Alberta immunization data standard. Version 1.6. Available from <https://open.alberta.ca/publications/alberta-immunization-data-standard-version-1-6>

Aa17: Alberta Government. Newborn metabolic screening minimum data standard. Version 3.5. Available from <https://open.alberta.ca/publications/newborn-metabolic-screening-minimum-data-standard-version-3-5>

Aa18: Alberta Government. Perinatal health program data standard. Version 1.1. Available from <https://open.alberta.ca/publications/perinatal-health-program-data-standard-version-1-1>

Aa19: British Columbia. Client Registry. Available from <https://www2.gov.bc.ca/gov/content/health/practitioner-professional-resources/health-information-standards/standards-catalogue/client>

Aa20: British Columbia. Provider Registry. Available from <https://www2.gov.bc.ca/gov/content/health/practitioner-professional-resources/health-information-standards/standards-catalogue/provider>

Aa21: British Columbia. Provider Laboratory Information Solution. Available from <https://www2.gov.bc.ca/gov/content/health/practitioner-professional-resources/health-information-standards/standards-catalogue/plis>

Aa22: British Columbia. Physician Information Technology Office. EMR-to-EMR Data Transfer & Conversion (E2E-DTC) Standard, Part II – Consolidated CDA Implementation Guide. Version 1.35. Available from <https://www2.gov.bc.ca/assets/gov/health/practitioner-pro/pito-e2e-dtc-part2-consolidated-implementation-guide.pdf>

Aa23: British Columbia. Conformance Standards Version 3 Pharmanet. Available from <https://www2.gov.bc.ca/assets/gov/health/practitioner-pro/software-development-guidelines/bc-conformance-standards-v3-pharmanet.zip>

Aa24. Shared Health Manitoba. EMR Certification. Baseline EMR Requirements Specification. Sep 13, 2019. Version 1.9. Available from <https://sharedhealthmb.ca/files/emr-certification-base-spec.pdf>

Aa25: Shared Health Manitoba. EMR Certification. eChart Manitoba Context-Sensitive Launch Interface Specification. Mar 31, 2019. Version 1.4. Available from <https://sharedhealthmb.ca/files/emr-certification-echart-spec.pdf>

Aa26: Shared Health Manitoba. EMR Certification. eHealth_hub – Laboratory Result Distribution Interface Specification. Mar 31, 2019. Version 1.4. Available from <https://sharedhealthmb.ca/files/emr-certification-hub-spec.pdf>

Aa27: Shared Health Manitoba. EMR Certification. eHealth_hub – Diagnostic Imaging Report Result Distribution Interface Specification. Mar 31, 2019. Version 1.4. Available from <https://sharedhealthmb.ca/files/emr-certification-hub-di-spec.pdf>

Aa28: Shared Health Manitoba. EMR Certification. eHealth_hub – Client Registry Query Service Interface Specification. Mar 31, 2019. Version 1.2. Available from <https://sharedhealthmb.ca/files/emr-certification-hub-crq-spec.pdf>

Aa29: Shared Health Manitoba. EMR Certification. Primary Care Quality Indicator Reminders and Data Extract Specification. Jun 16, 2020. Version 4.1. Available from <https://sharedhealthmb.ca/files/emr-certification-pc-spec.pdf>

Aa30: Shared Health Manitoba. EMR Certification. Interprofessional Team Data Extract Specification. Mar 31, 2020. Version 1.1. Available from <https://sharedhealthmb.ca/files/emr-certification-de-spec.pdf>

Aa31: Newfoundland & Labrador Centre for Health Information. Provincial Discharge Abstract Database (PDADP) User Guide v.1.0, Aug 2017. Available from <https://www.nlchi.nl.ca/images/2016-17_PDAD_User_Guide_v1.0_2017-08-08.pdf>

Aa32: Newfoundland & Labrador Centre for Health Information. Live Birth System 2016 Master Methodology. Aug 2017 v.1.0. Available from <https://www.nlchi.nl.ca/images/2016_NLCHI_LBS_User_Guide_Final.pdf>

Aa33: Newfoundland & Labrador Centre for Health Information. Mortality System Data User Guide. Aug 2017 v.1.0. Available from <https://www.nlchi.nl.ca/images/2016_NLCHI_Mortality_User_Guide_Final.pdf>

Aa34: Newfoundland & Labrador Centre for Health Information. Stillbirth System 2016 User Guide. Aug 2017 v.1.0. Available from <https://www.nlchi.nl.ca/images/2016_NLCHI_Stillbirth_User_Guide_Final.pdf>

Aa35: eHealth Ontario Acute and Community CDR Input Standard Version 2.5.1, Aug 23, 2018. Available from <https://ehealthontario.on.ca/en/standards/acute-and-community-cdr-input-standard>

Aa36: Cancer Care Ontario. Ontario Oncology Information System Standards: Defining its Meaningful Use, Mar 31, 2014. Available from <https://www.cancercareontario.ca/sites/ccocancercare/files/guidelines/full/OncInfoSysStandards_0.pdf>

Aa37: eHealth Ontario. Diagnostic Imaging Common Service Standard (DI CS). Available from <https://ehealthontario.on.ca/en/standards/diagnostic-imaging-common-service-standard-di-cs>

Aa38: eHealth Ontario. Ontario Laboratories Information System (OLIS) Standard. Available from <https://ehealthontario.on.ca/en/standards/ontario-laboratories-information-system-standard>

Aa39: eHealth Ontario. Provincial Client Registry (PCR) Standards Release 2 (R2). Available from <https://ehealthontario.on.ca/en/standards/provincial-client-registry-standards-release-2-r2>

Aa40: eHealth Ontario. Provincial Clinical Document Architecture Header Standard. Available from <https://ehealthontario.on.ca/en/standards/provincial-clinical-document-architecture-header-standard>

Aa41: eHealth Ontario. Provincial Provider Registry Specification. Available from <https://ehealthontario.on.ca/en/standards/provincial-provider-registry-specification>

Aa42: eHealth Ontario. Digital Health Drug Repository Specification – FHIR. Available from <https://ehealthontario.on.ca/en/standards/digital-health-drug-repository-specification-fhir>

Aa43: eHealth Ontario. Digital Health Drug Repository Specification – FHIR (Release 3). Available from <https://ehealthontario.on.ca/en/standards/digital-health-drug-repository-specification-fhir-release-3>

Aa44: eHealth Ontario. Digital Health Immunization Repository Consumer Access Specification – FHIR. Available from <https://ehealthontario.on.ca/en/standards/digital-health-immunization-repository-consumer-access-specification-fhir>

Aa45: eHealth Ontario. Digital Health Immunization Repository Specification – FHIR (Release 1). Available from <https://ehealthontario.on.ca/en/standards/digital-health-immunization-repository-specification-fhir-release-1>

Aa46: eHealth Ontario. Digital Health Immunization Repository Specification – FHIR (Release 4). Available from <https://ehealthontario.on.ca/en/standards/digital-health-immunization-repository-specification-fhir-release-4>

Aa47: eHealth Ontario. Provincial Client Registry – HL7 FHIR Implementation Guide. Available from <https://ehealthontario.on.ca/en/standards/provincial-client-registry-hl7-fhir-implementation-guide>

Aa48: eHealth Ontario. Provincial Provider Registry. Available from <https://ehealthontario.on.ca/en/standards/provincial-provider-registry-fhir>

Aa49: OntarioMD. EMR Specifications Library – EMR Core Data Set Version 5.0, Aug 2017. Available from <https://www.ontariomd.ca/emr-certification/emr-specification/library>

Aa50: OntarioMD. EMR Specifications Library – EMR Hospital Report Manager (HRM) Version 4.3, Aug 2017. Available from <https://www.ontariomd.ca/emr-certification/emr-specification/library>

1. EHRs and Standards - Entities in Canada (may be organizations, committees or initiatives)

| Entity | EHR System and Standard | Source* | Type |
| --- | --- | --- | --- |
| Canada Health Infoway (Infoway) (2) | PrescribeIT  Terminology Gateway | Ab01  Ab02 | S  S |
| Canadian Institute for Health Information (CIHI) | ***CIHI Data Holdings****  Discharge Abstract Database, 2018-2019  National Ambulatory Care Reporting System: Clinic Lite Web Entry Tool User Guide, 2018-2019  Hospital Morbidity Database , 2018-2019  Hospital Mental Health Database, Data Dictionary for Fiscal Year, 2018-2019  Canadian Joint Replacement Registry Minimum Data Set Data Elements, 2018-2019  National Rehabilitation Reporting System –Rehabilitation Minimum Data Set Manual, Feb 2016  Ontario Mental Health Reporting System, 2018-2019  Home Care Reporting System, 2018-2019  Continuing Care Reporting System, 2018-2019  Canadian Organ Replacement Register, 2018  National Trauma Registry Comprehensive Data Set-Data Dictionary, 2012-2013  Ontario Trauma Registry Comprehensive Data Set Data Dictionary, 2018-2019  Primary Health Care Electronic Medical Record Content Standard, 2014  National Prescription Drug Utilization Information System, 2018-2019  National Physician Database, 2017-2018  Scott’s Medical Database, 2018  Health Workforce Database Record-level Data, 2017-2018  Health Workforce Database Aggregate-level Data, 2017-2018  Commonwealth Fund, 2019  Canadian Patient Experiences Reporting System, 2018-2019  Patient Reported Outcome Measures, 2019  Integrated interRAI Reporting System, 2018-2019  CIHI Reference Data Model Toolkit, 2019  In Pursuit of Health Equity: Defining Stratifiers for Measuring Health Inequity, April 2018, p20 | Ab0x  Ab03  Ab04  Ab05  Ab06  Ab07  Ab08  Ab09  Ab10  Ab11  Ab12  Ab13  Ab14  Ab15  Ab16  Ab17  Ab18  Ab19  Ab20  Ab21  Ab22  Ab23  Ab24  Ab25  Ab26 | D  D  D  D  D  D  D  D  D  D  D  D  S  D  D  D  D  D  D  D  D  D  S  S |
| Canadian Primary Care Sentinel Surveillance Network (CPCCSN) (1) | CPCSSN Data Dictionary, 2015 | Ab27 | D |
| Statistics Canada (StatCan)  (1 – database, i.e. CCR)  (6 – definitions) | Canadian Cancer Registry (CCR) – Variables -> Sex of person category  Classification of Sex, approved Jan 25, 2018  Variant of Classification of Sex, approved Jan 25, 2018  Classification of Gender, Approved Jan 25, 2018  Classification of Cisgender, approved Jan 25, 2018  Classification of Transgender, approved Jan 25, 2018  Preparing Gender Identity for the National Statistical System, May 2018 | Ab28  Ab29  Ab30  Ab31  Ab32  Ab33  Ab34 | D  S  S  S  S  S  S |
| Centre for Addictions and Mental Health (CAMH) | Barbara Am, Chaim G & Doctor F. Asking the Right Questions 2 – Talking with clients about sexual orientation and gender identity in mental health, counselling and addiction settings. 2007 | Ab35 | S |
| Tri-Hospital + Toronto Public Health | We Ask Because We Care. The Tri-Hospital + TPH Health Equity Data Collection Research Project Report. Summary Report, June 2013 | Ab36 | S |
| Canadian Longitudinal Study on Aging (CSLA) | DataPreview Portal Dataset Search | Ab37 | D |

Legends: S-Standard specifications, D-Database repository

*Source –CIHI website of all Data Holdings. CIHI staff validated all of the information sources shown in this table under CIHI.

Reference Sources: EHRs and Standards - Entities in Canada

Ab01. Canada Health Infoway. Gender of people. Available from <https://tgateway.infoway-inforoute.ca/singlesubset.html?id=2.16.840.1.113883.2.20.3.447> (login to InfoCentral required)

Ab02. Canada Health Infoway. Administrativegender. Available from <https://tgateway.infoway-inforoute.ca/html/singlesubset.html?id=2.16.840.1.113883.2.20.3.308&versionid=20140930> (login to InfoCentral required)

Ab0x: Canadian Institute for Health Information (CIHI). Data Holdings. Available from <https://www.cihi.ca/en/access-data-and-reports/make-a-data-request/data-holdings>

Ab27: Canadian Primary Care Sentinel Surveillance Network. CPCSSN Data Dictionary 2015. Available from <https://cpcssn.ca/wp-content/uploads/2015/02/CPCSSN-Data-Dictionary.pdf>

Ab28: Statistics Canada. Canadian Cancer Registry – Sex of person, category. Available from <https://www23.statcan.gc.ca/imdb/p2SV.pl?Function=assembleDESurv&DECId=467214&RepClass=591&Id=1211637&DFId=180443>

Ab29: Statistics Canada. Classification of sex. Available from <https://www23.statcan.gc.ca/imdb/p3VD.pl?Function=getVD&TVD=469273>

Ab30: Statistics Canada. Variant of classification of sex. Available from <https://www23.statcan.gc.ca/imdb/p3VD.pl?Function=getVD&TVD=469276>

Ab31: Statistics Canada. Classification of gender. Available from <https://www23.statcan.gc.ca/imdb/p3VD.pl?Function=getVD&TVD=467245>

Ab32: Statistics Canada. Classification of cisgender and transgender. Available from <https://www23.statcan.gc.ca/imdb/p3VD.pl?Function=getVD&TVD=469267&CVD=469268&CPV=C&CST=25012018&CLV=1&MLV=2>

Ab33: Statistics Canada. Classification of cisgender and transgender. Available from <https://www23.statcan.gc.ca/imdb/p3VD.pl?Function=getVD&TVD=469267&CVD=469268&CPV=T&CST=25012018&CLV=1&MLV=2>

Ab34: Statistics Canada. Preparing Gender Identity for the National Statistical System, May 2018. Available from <http://www.asi-iea.ca/en/files/2018/09/Placemat_gender_ID_May12.pdf>

Ab35: Centre for Addictions and Mental Health (CAMH). Asking the Right Questions 2. Available from <https://www.porticonetwork.ca/documents/489955/0/Asking+the+right+questions+2/80d19139-b361-4547-989d-e5b6479651b0>

Ab36: Tri-Hospital+Toronto Public Health. We ask because we care, The Tri-Hospital + TPH Health Equity Data Collection Research Project Report– Summary Report Jun 2013. Available from <https://www.porticonetwork.ca/documents/43843/277776/FINAL+Summary+Report+June+2013.pdf/2fc7d2ff-6573-427b-a247-9b347b1d55d9>

Ab37: Canadian Longitudinal Study on Aging (CSLA). Gender of person who participant provided most care giving assistance. Available from <https://datapreview.clsa-elcv.ca/mica/variable/com%3ACAG_GNDR_COM%3ACollected#/>

1. Health Data Exchange and Terminology Standards – Standards Communities (may be organizations, committees, initiatives or government agencies involved with health information standards)

| Community | Health Information Standard | Code System | Data Element Name | Source |
| --- | --- | --- | --- | --- |
| HL7 | Version 2.6 Table 0001 | HL7V2-Table 0001 | Administrative Sex | Ac01 |
|  | Version 3 | HL7V3 Administrative Gender | Administrative Gender | Ac02 |
|  | FHIR Release 4.0.1 | HL7V3 Administrative Gender | Administrative Gender | Ac03 |
|  | FHIR Release 4.0.1 (draft for comment) | HL7V3 Gender Identity | Gender Identity | Ac27 |
|  | FHIR U.S. Core Profiles Release 4.0.1 | HL7 FHIR –V3 Administrative Gender | gender | Ac04 |
|  | FHIR U.S. Core Profiles Release 3.1.1 | HL7 FHIR –V3 Administrative Gender, NullFlavor | us-core-birthsex | Ac05 |
|  | FHIR UK Core Patient Profile | HL7 FHIR –V3 Administrative Gender | gender | Ac06 |
|  | NHS Data Model and Dictionary Version 3 | PersonStatedGenderCode | PersonStatedGenderCode | Ac07 |
|  | Gender Harmony Project | Context definition names only - TBA | Gender Identity | Ac09 |
|  |  |  | Recorded Gender or Sex |  |
|  |  |  | Sex for Clinical Use |  |
| DICOM | Registry of DICOM Data Elements, 2013 | Tag (0010,0040) | Patient’s Sex | Ac10 |
|  | PS3.16 2020 Content Mapping Resources | CID 7455 Sex | Patient’s Sex | Ac11 |
|  | PS3.16 Correction Proposal (CP) | CP1927_14 Tag (0010,0040) | Patient’s Sex | Ac12 |
|  |  | CP1927_14 Tag (0010,xxxx) | Patient’s Gender |  |
| ONC | Interoperability Standards Advisory (ISA) 2020 | LOINC, SNOMED CT, HL7V3 | Patient Gender Identity | Ac13 |
|  |  | LOINC, HL7V3 | Patient Sex (At Birth) | Ac14 |
| NHS | NHS Data Model and Dictionary | NHS Data Dictionary Version 3 | Gender Identity Code (Sexual Health) | Ac16 |
|  |  |  | Gender Identity Same at Birth Indicator | Ac17 |
|  |  |  | Sex of Patients | Ac18 |
| ISO | ISO/IEC5218 | Codes for Human Sexes | Sex | Ac20 |
| OpenEHR | OpenEHR Clinical Knowledge Manager | Gender Archetype | Gender | Ac21 |
| BioPortal | Repository of biomedical ontologies, 2020 | Gender, Sex and Sexual Orientation Ontology | Gender | Ac22 |
|  |  |  | Sex |  |
| AIHW | Metadata Online Repository (METeOR) | AIHW | Gender | Ac23 |
|  |  |  | Sex | Ac24 |
| LOINC | LOINC, Version 2.65 | LOINC | Sex, Gender | Ac25 |
| SNOMED CT | SNOMED CT, Release 2020-03-09 | SNOMED CT | Sex, Gender | Ac26 |

Legends: HL7 – Health Level Seven; DICOM - Diagnostic Imaging and Communication Standard; NHS – National Health Services; ONC - Office of National Coordinator for Health IT; AIHW – Australia Institute of Health and Welfare

Reference Sources: Health Data Exchange and Terminology Standards

Ac01: HL7 Version 2.6. Data Definition Tables – Table 0001 Administrative Sex. Available from <https://www.hl7.org/special/committees/vocab/V26_Appendix_A.pdf>

Ac02: HL7 Version 3. AdministrativeGender. Available from <http://www.hl7.org/v3ballotarchive/v3ballot/html/infrastructure/vocabulary/AdministrativeGender.html>

Ac03: HL7 FHIR Release 4.0.1 AdministrativeGender. Available from <https://www.hl7.org/fhir/v3/AdministrativeGender/vs.html>

Ac04: HL7 FHIR U.S. Core Profiles Release 4.0.1 Gender. Available from <http://hl7.org/fhir/R4/valueset-administrative-gender.html>

Ac05: HL7 FHIR U.S. Core Profiles Release 3.1.1. us-core-birthsex. Available from <http://hl7.org/fhir/us/core/StructureDefinition-us-core-birthsex.html>

Ac06: HL7 FHIR UK Core Patient Profile – UK Core Person Stated Gender Code. Available from <https://simplifier.net/guide/ukcoredevelopment/allvaluesets#ValueSetUKCorePersonStatedGenderCode>

Ac07: HL7 NHS Data Model and Dictionary. Person Stated Gender Code. Available from <https://datadictionary.nhs.uk/attributes/person_stated_gender_code.html>

Ac09: HL7 Gender Harmony Project. Gender Harmony Context Definitions. Available from <https://confluence.hl7.org/display/VOC/Gender+Harmony+Context+Definitions>

Ac10: DICOM. C.2.3 Patient Demographic Module - Patient’s Sex. Available from <http://dicom.nema.org/dicom/2013/output/chtml/part03/sect_C.2.html>

Ac11: DICOM. PS3.16.2020 Content Mapping Resources, CID 7455 Sex. Available from <http://dicom.nema.org/medical/dicom/current/output/html/part16.html#sect_CID_7455>

Ac12: DICOM. PS3.16 Correction Proposal, CP1927_14 Tag Patient’s Sex and Patient’s Gender. Available from <https://confluence.hl7.org/download/attachments/40743893/cp1927_14_Patient_sex_and_gender.docx?api=v2>

Ac13: ONC. ISA 2020. Patient Gender Identity. Available from <https://www.healthit.gov/isa/representing-patient-gender-identity>

Ac14: Office of National Coordinator (ONC). Interoperability Standards Advisory (ISA) 2020. Patient Sex (At Birth). Available from <https://www.healthit.gov/isa/representing-patient-sex-birth>

Ac16: National Health Services (NHS). NHS Data Model and Dictionary – Gender Identity Code for Sexual Health. Available from <https://datadictionary.nhs.uk/attributes/gender_identity_code_for_sexual_health.html>

Ac17: National Health Services (NHS). NHS Data Model and Dictionary – Gender Identity Same at Birth Indicator. Available from <https://datadictionary.nhs.uk/attributes/gender_identity_same_at_birth_indicator.html>

Ac18: National Health Services (NHS). NHS Data Model and Dictionary – Sex of Patient. Available from <https://datadictionary.nhs.uk/attributes/sex_of_patients.html>

Ac20: Wikipedia. ISO/IEC 5218 Information Technology – Codes for the representation of human sexes. Available from <https://en.wikipedia.org/wiki/ISO/IEC_5218>

Ac21: OpenEHR. Gender Archetype. Available from <https://ckm.openehr.org/ckm/archetypes/1013.1.3715/mindmap>

Ac22: BioPortal – Gender, Sex and Sexual Orientation Ontology – Gender and Sex. Available from <https://bioportal.bioontology.org/ontologies/GSSO/?p=classes&conceptid=root>

Ac23: Australian Institute of Health and Welfare (AIHW). Metadata Online Registry (METeOR) - Gender. Available from <https://meteor.aihw.gov.au/content/index.phtml/itemId/635994>

Ac24: Australian Institute of Health and Welfare (AIHW). Metadata Online Registry (METeOR) - Sex. Available from <https://meteor.aihw.gov.au/content/index.phtml/itemId/635126>

Ac25: LOINC. Sex and Gender. Available from <https://search.loinc.org/searchLOINC/search.zul?query=sex> and <https://search.loinc.org/searchLOINC/search.zul?query=gender> (login to LOINC search required)

Ac26: SNOMED International. Sex and Gender. Available from <https://browser.ihtsdotools.org/>?

Ac27: HL7 FHIR R4 Draft for Comment. Gender identity. Available from <https://hl7.org/fhir/2018Jan/codesystem-gender-identity.html>
