# Supplementary material for: An Environmental Scan of Sex and Gender in Electronic Health Records: Analysis of Public Information Sources
Source: J Med Internet Res. 2020 Nov 11;22(11):e20050. doi: 10.2196/20050 (PMC7688387; doi:10.2196/20050)
Supplement: Multimedia Appendix 2 [file jmir_v22i11e20050_app2.docx]

Appendix 2 - Existing sex definitions in Canadian EHRs.

| Data Element | Code System | Value Set | Source* |
| --- | --- | --- | --- |
| Administrative Sex | HL7-0001, V2  HL7V2-0001  HL7V2-0001  MB | M-Male, F-Female, U-Unknown  M-Male, F-Female, UNK-Unknown, UN-Undifferentiated  M-Male, F-Female, U-Unknown/Undifferentiated, O-Other  M-Male, F-Female, U-Undetermined, Unknown or Other | Aa08, Aa39v2  Aa28  Aa35  Aa24, Aa29, Aa30 |
| Sex | HL7V2  NLCHI  NLCHI  CIHI  StatCan  CIHI  CIHI  CPCSSN  CIHI  CIHI  CIHI | *F-Female, M-Male, U-Unknown*  1-Male, 2-Female  1-Male, 2-Female, 3-Unknown  1-Male, 2-Female, U-Unknown  M-Male, F-Female  1-Male, 2-Female, O-Other  1-Male, 2-Female, O-Other (includes hermaphrodites, transsexual)  Male, Female  M-Male, F-Female, U-Unknown, O-Other  M-Male, F-Female, U-Undifferentiated, 9-Not Stated  M-Male, F-Female, UN-Not assigned male or female  155939-Female, 133338-Male, 281-Null, 39-Unknown, 1-Undifferentiated | Aa39  Aa34  Aa33  Ab14  Ab28  Ab06  Ab08  Ab27  Ab16  Ab20  Ab24 |
| Sex Code | CIHI  CIHI | M-Male, F-Female  1-Male, 2-Female, 3-Other | Ab13  Ab19 |
| Sex (Biological Sex) | CIHI  CIHI  CIHI  CIHI | M-Male, F-Female, O-Other (person could not be uniquely identified as male or female, e.g. hermaphrodite)  M-Male, F-Female, O-Other  M-Male, F-Female, O-Other (trans-sexual, hermaphrodite)  M-Male, F-Female, Blank (if unknown) | Ab11  Ab09  Ab12  Ab10 |
| Patient’s Sex | DICOM (0010,0040) | *M-Male, F-Female, O-Other* | Aa37 |
| Sex assigned at Birth | CIHI | M-Male, F-Female, I-Indeterminate, UNK-Unknown | Ab23 |
| Infant’s Sex | NLCHI | *1-Male, 2-Female, 3-Unknown* | Aa32 |

Legends: MB-Manitoba, StatCan-Statistics Canada, DICOM-Diagnostic Imaging and Communication, NLCHI-Newfoundland & Labrador Centre for Health Information, CPCSSN-Canadian Primary Care Sentinel Surveillance Network; grey entries in italics are duplicates already accounted for in another value set
