# Supplementary material for: An Environmental Scan of Sex and Gender in Electronic Health Records: Analysis of Public Information Sources
Source: J Med Internet Res. 2020 Nov 11;22(11):e20050. doi: 10.2196/20050 (PMC7688387; doi:10.2196/20050)
Supplement: Multimedia Appendix 3 [file jmir_v22i11e20050_app3.docx]

Appendix 3 - Existing gender definitions in Canadian EHRs.

| Data Element | Code System | Value Set | Source* |
| --- | --- | --- | --- |
| Administrative Gender Type | HL7V2, V3 | F-Female, M-Male, U-Unknown, O-Other, I-Indeterminate | Aa05 |
| Administrative Gender | HL7V3,CDA  HL7V3  HL7V2  HL7-FHIR | F-Female, M-Male, UN-Undifferentiated  F-Female, M-Male, U-Unknown  F-Female, M-Male, UN-Undifferentiated, UNK-Unknown  Male-Male, Female-Female, Other-Other, Unknown-Unknown | Aa19, Aa22, Aa40, Ab02  Aa20  Aa21  Ab01 |
| Administrative Gender Code | AB  HL7V3 | *F-Female, M-Male, U-Unknown, O-Other, I-Indeterminate*  *F-Female, M-Male, UN-Undifferentiated* | Aa14  Aa37 |
| Clinical Gender | HL7V2 | *F-Female, M-Male, UN-Undifferentiated, UNK-Unknown* | Aa21 |
| Gender | HL7V2, AB  HL7,V3,AB  MN  HL7V2-0001  ND  HL7-FHIR  CIHI  CIHI  CSLA  CIHI  CIHI | F-Female, M-Male, U-Unknown, O-Other  *F-Female, M-Male, U-Unknown*  F-Female, M-Male  F-Female, M-Male, UNK-Unknown, UN-Undifferentiated  F-Female, M-Male, U-Undifferentiated stillbirth only, O-Other (trans-sexual or hermaphrodite)  Female-Female, Male-Male, Unknown-Unknown, Other-Other  F-Female, M-Male, U-Undifferentiated, stillbirths only, O-Other or unknown  1-Male, 2-Female, ZZ-Other  1-Male, 2-Female, 8-Don’t know/No answer, 9-Refused  Female-Female, Male-Male, Unknown-Unknown  Female-Female, Male-Male, Refused or Something Else | Aa06, Aa13, Aa15, Aa18, Aa49, Ab22  Aa07, Aa10, Aa39v3, Aa50  Aa25  Aa26, Aa27, Aa28  Aa31  Aa42, Aa43, Aa44, Aa45, Aa46, Aa47, Aa48, Ab01  Ab03, Ab04  Ab07  Ab37  Ab18  Ab21 |
| Gender Code | HL7  AB  HL7V3  CIHI  CIHI  CIHI | *F-Female, M-Male, U-Unknown, O-Other*  F-Female, M-Male, I-Undifferentiated stillbirth only, U-Unknown, O-Other; for trans-sexual or hermaphrodite, *U-Undifferentiated; for stillbirths only (discontinued)*  *F-Female, M-Male*  F-Male, M-Male, O-Other (Trans-sexual or hermaphrodites)  1-Male, 2-Female, 3-Unknown  F-Female, M-Male, 7-Not Collected, 9-Unknown | Aa04, Aa12, Aa16  Aa02  Aa41  Ab05  Ab17  Ab19 |
| Patient Gender | HL7  HL7V3 | *F-Female, M-Male, U-Unknown*  F-Female, M-Male, UN-Undifferentiated | Aa09, Aa10, Aa23  Ab15 |
| Patient Gender Code | AB | *F-Female, M-Male, U-Unknown* | Aa11 |
| Person Gender | AB | *F-Female, M-Male, UN-Undifferentiated* | Aa03 |
| Gender Identity | CIHI  CIHI | F-Female, M-Male, OTH-Other gender identity, UNK-Not Known, NA-Not Applicable  F-Female, M-Male, D-Gender Diverse, UNK-Not Known, NA-Not Applicable | Ab24  Ab23 |
| Newborn Gender | HL7V2 | *F-Female, M-Male, U-Unknown, O-Other* | Aa17 |
| Lab Newborn Gender |  |  |  |

Legends: AB-Alberta, CLSA-Canadian Longitudinal Study on Aging, CIHI, DICOM; grey entries in italics are duplicates already accounted for in another value set
