# Supplementary material for: An Environmental Scan of Sex and Gender in Electronic Health Records: Analysis of Public Information Sources
Source: J Med Internet Res. 2020 Nov 11;22(11):e20050. doi: 10.2196/20050 (PMC7688387; doi:10.2196/20050)
Supplement: Multimedia Appendix 4 [file jmir_v22i11e20050_app4.docx]

Appendix 4 - Expanded sex and gender definitions in Canadian health organizations.

| Organization | Concept | Value Set (with description/definition if available) | Source* |
| --- | --- | --- | --- |
| Statistics Canada | Sex | M-Male, F-Female | Ab29 |
|  | Variant of Sex | M-Male, F-Female, I-Intersex | Ab30 |
|  |  | Male includes persons who were reported as being of male sex, assigned at birth |  |
|  |  | Female includes persons who were reported as being of female sex, assigned at birth |  |
|  |  | Intersex includes persons who were reported as being of intersex, assigned at birth |  |
|  | Gender | M-Male gender, F-Female gender, D-Gender diverse | Ab31 |
|  |  | Female gender includes persons whose current gender was reported as female. This includes cisgender and transgender persons who were reported as being female |  |
|  |  | Male gender includes persons whose current gender was reported as male. This includes cisgender and transgender persons who were reported as being male |  |
|  |  | Gender diverse includes persons whose current gender was not reported exclusively as male or female. It includes persons who were reported as being unsure of their gender, persons who were reported as both male and female, or neither male nor female |  |
|  | Cisgender | C-Cisgender, CM-Cisgender man, CF-Cisgender woman | Ab32 |
|  |  | This category includes persons who have reported that their sex assigned at birth is the same as their current gender |  |
|  | Transgender | T-Transgender, TM-Transgender man, TF-Transgender woman, TG-Transgender person, n.e.c | Ab33 |
|  |  | This category includes persons whose sex assigned at birth was reported as female and whose current gender was reported as other than female; or persons whose sex assigned at birth was reported as male and whose current gender was reported as other than male. It also includes persons who were reported as being unsure of their gender or persons who were reported as both male and female, bi-gender or neither male nor female |  |
|  | Nonbinary Gender | Trans-Masculine Demi-Boy, Trans-Feminine Demi-Girl, Pan-Gender Poly-Gender Bi-Gender Two-Spirit, Gender-Fluid Neutrois, Genderless, Agender | Ab34 |
| Canadian Institute for Health Information | Sex at Birth | F-Female, M-Male, I-Indeterminate, UNK-Unknown | Ab25,p59 |
|  | Gender | F-Female, M-Male, D-Gender Diverse, UNK-Unknown, NA-Not Applicable |  |
|  | Gender Identity | A person’s internal and experience, a sense of being a woman, man, both, neither or along a spectrum |  |
|  | Lived Gender | How a person publicly presents their gender, which include behavior, appearances, name and pronoun |  |
|  | Intersex | Refers to a variety of conditions where a person has atypical development of sex characteristics, such as reproductive anatomy, sex chromosomes or sex-related hormones, that is not consistent with typical definitions of male of female | Ab26,p16 |
| Centre for Addictions Mental Health | Gender Identity | Female, Male, Transsexual, Transgender, Genderqueer, Two-Spirit, FTM (Female-to-male), MTF (male-to-female), Intersex, Unsure, Questioning, Other, Prefer not to answer | Ab35 |
| Tri-Hospital + Toronto Public Health | Gender (check one) | Female, Male, Trans-Female to Male, Trans-Male to Female, Intersex, Other (specify), Prefer not to answer, Do not know | Ab36 |
