# Supplementary material for: An Environmental Scan of Sex and Gender in Electronic Health Records: Analysis of Public Information Sources
Source: J Med Internet Res. 2020 Nov 11;22(11):e20050. doi: 10.2196/20050 (PMC7688387; doi:10.2196/20050)
Supplement: Multimedia Appendix 5 [file jmir_v22i11e20050_app5.docx]

Appendix 5 - Published definitions for sex-related concepts from standards communities.

| Community | Data Element | Code System | Value Set (with description/definition if available) | Source |
| --- | --- | --- | --- | --- |
| HL7  Version 2.6 | Administrative Sex | V2 Table 0001 | A-Ambiguous, F-Female, M-Male, N-Not Applicable, O-Other, U-Unknown | Ac01, p24 |
| HL7 FHIR US Core Release 3.1.0 | us-core-birthsex | V3 Administrative Gender    V3 NullFlavor | F-Female, M-Male, UNK-Unknown  Definition: UNK - A proper value is applicable, but not known. Usage Notes: This means the actual value is not known. If the only thing that is unknown is how to properly express the value in the necessary constraints (value set, datatype, etc.), then OTH or UNC flavor should be used. No properties should be included for a datatype with this property unless: Those properties themselves directly translate to a semantic of "unknown". (E.g. a local code sent as a translation that conveys 'unknown') Those properties further qualify the nature of what is unknown. (E.g. specifying a use code of "H" and a URL prefix of "tel:" to convey that it is the home phone number that is unknown.) | Ac05 |
| HL7 Gender Harmony Project | Sex for Clinical Use | Context definition names only, To Be Announced, as of Feb 13, 2020 | Male, Female, Non-binary, Unknown  From observable info, Types –Chromosomal sex, Anatomic sex, Sex for imaging, Hormonal/Organ status sex | Ac09 |
| DICOM | Patient’s Sex | Tag (0010,0040) | M-male, F-female, O-other | Ac10 |
|  | Sex | Table CID7455 | M-Male, F-Female, U-Unknown sex, MP-Male Pseudohermaphrodite, FP-Female Pseudohermaprodite, H-Hermaphrodite, MC-Male changed to Female, FC-Female changed to Male, 121104-Ambiguous sex, 12102-Other sex, 121103-Undetermined sex | Ac11 |
|  | Patient’s Sex | Tag (0010,0040) Proposed | M-male, F-female, O-non-binary, e.g. intersex, other situations where neither male nor female apply clinically | Ac12 |
| ONC ISA 2020 | Patient Sex (At Birth) | LOINC  V3 NullFlavor | 76689-9 Sex assigned at birth  M-Male, F-Female, UNK-Unknown | Ac14 |
| NHS | Sex of Patients | NHS Data Dictionary Version 3, Dec 2019 | 1-Male, 2-Female, 8-Not specified, 9-Home Leave (for ward operational plans only) | Ac18 |
| ISO/IEC | Sex | ISO/IEC 5218 | 0-Not known, 1-Male, 2-Female, 9-Not applicable | Ac20 |
| OpenEHR | Sex assigned at birth | Gender Archetype | Anatomical characteristics observed and registered at birth, e.g. Male, Female, Intersex | Ac21 |
| BioPortal | Sex | Gender, Sex, and Sexual Orientation Ontology | Biological sex, Intersex, Sex at birth | Ac22 |
|  | Biological sex |  | Anatomical sex, Brain sex, Genotypic sex, Hormonal sex, Indeterminate sex, phenotypic sex |  |
|  | Anatomic sex |  | Gonadal sex |  |
|  | Genotypic sex |  | Chromosomal sex, Genetic sex |  |
| AIHW | Sex | METeOR | 1-Male, 2-Female, 3-Other, 9-Not stated/inadequately described; alternate scheme M-Male, F-Female, X-Other | Ac24 |
| LOINC | Sex | LOINC, Version 2.65 | 76689-9 Sex assigned at birth  46098-0 Sex  11883-6 Fetal Narrative Sex US, 11882-8 Fetal Sex US  21840-4 Sex NAACCR v.11, 74698-2 Sex [AHRQ]  72143-1 Sex [HL7.v3], 54131-8 Sex [USSG-FHT] | Ac25 |
| SNOMED CT | Sex | SNOMED CT, July 2019 Release | Examples only: 184100006\|Patient sex\|, 268476009\|Sex of baby\|, 734000001\|Biological Sex\|, 302081005\|Finding of sex of baby\|, 248153007\|Male\|, 248152002\|Female\|, 32570691000036108\|Intersex\| | Ac26 |

**Legends:** HL7-Health Level Seven, FHIR-Fast Healthcare Interoperability Resources, DICOM-Diagnostic Imaging & Communication, ONC ISA-Office of National Coordinator Interoperability Standards Advisory, NHS-National Health Services, ISO-International Standards Organization, AIHW-Australia Institute of Health and Welfare, LOINC-Logical Observations Identifiers Names and Codes, SNOMED CT-SNOMED Clinical Terms
