# Supplementary material for: An Environmental Scan of Sex and Gender in Electronic Health Records: Analysis of Public Information Sources
Source: J Med Internet Res. 2020 Nov 11;22(11):e20050. doi: 10.2196/20050 (PMC7688387; doi:10.2196/20050)
Supplement: Multimedia Appendix 6 [file jmir_v22i11e20050_app6.docx]

Appendix 6 - Published definitions for gender-related concepts from standards communities.

| Community | Data Element | Code System | Value Set (with description/definition if available) | Source |
| --- | --- | --- | --- | --- |
| HL7 V3 | Administrative Gender | V3 Administrative Gender | F-Female, M-Male, UN-Undifferentiated  Description: The gender of a person used for administrative purposes (as opposed to clinical gender)  Definition: UN - The gender of a person could not be uniquely defined as male or female, such as hermaphrodite | Ac02 |
| HL7 FHIR Version 4.0.1 | Administrative Gender | V3 Administrative Gender | F-Female, M-Male, UN-Undifferentiated  Definition: UN - The gender of a person could not be uniquely defined as male or female, such as hermaphrodite | Ac03 |
| HL7 FHIR Version 4.0.1 | Gender Identity | Gender Identity | Transgender-female, transgender-male, non-binary, male, female, other, non-disclose | Ac27 |
| HL7 FHIR U.S. Core Patient Profile | gender | V3 Administrative Gender | Male-Male, Female-Female, Other-Other, Unknown-Unknown  Definition: The gender of a person used for administrative purposes | Ac04 |
| HL7 FHIR U.K. Core  Patient Profile | Gender | V3 Administrative Gender | Female-Female, Male-Male, Unknown-Unknown, Other-Other | Ac06 |
| NHS | PersonStated GenderCode | PersonStated GenderCode | 1-Male, 2-Female, 9-Indeterminate (unable to be classified as either male or female), X-Not known (Not recorded) | Ac07, Ac08 |
| HL7  Gender Harmony Project | Gender Identity | Context definition names only, To Be Announced, as of Feb 13, 2020 | Examples – Man, Woman, Boy, Girl, Non-binary, NotExpressed | Ac09 |
|  | Recorded Gender or Sex Identity (Legal Gender Previously) |  | Aligned with Administrative Sex/Gender, should be qualified by Identity Document Type, Time Frame, Jurisdiction/Organization, e.g. passport, driver’s license, birth certificate, sex assigned at birth |  |
| DICOM | Patient’s Gender | Tag (0010,xxxx) Proposed | To be revised when HL7 Vocab reaches consensus | Ac12 |
| ONC ISA2020 | Patient Gender Identity | LOINC  SNOMED CT (SCT)  HL7 V3 | 77691-5 Gender Identity  446151000124109\|Male\|, 446141000124107\|Female\|  407377005\|Female-to-Male (FTM)/Transgender Male/ Trans Man\|, 407376001\|Male-to-Female (MTF)/ Transgender Female/Trans Woman\|  446131000124102\|Identifies as non-confirming gender (US synonyms include –Genderqueer; identifies as neither exclusively male nor female, non-binary gender)\|  OTH-Additional gender category or other, please specify  ASKU-Choose not to disclose | Ac13 |
| NHS | Gender Identity Code (Sexual Health) | NHS Data Dictionary Version 3, Dec 2019 | 1-Male (including trans man), 2-Female (including trans woman), 3-Non-binary, 4-Other (not listed), Z-Not Stated (person asked but declined to provide a response) | Ac16 |
|  | Gender Identity Same at Birth Indicator |  | Y-Yes – the person’s gender identity is the same as their gender assigned at birth, N- No – the person’s gender identity is not the same as their gender assigned at birth, Z- Not Stated (person asked but declined to provide a response) | Ac17 |
| OpenEHR | Administrative gender | Gender Archetype | For administrative use, e.g. Male, Female, Other. Aligns with HL7 FHIR Person.gender | Ac21 |
|  | Legal gender |  | For official/legal use, e.g. birth certificate, passport |  |
|  | Gender expression |  | Expression of gender by individual as demonstrated by behavior, speech, clothes or other external characteristics, e.g. Masculine, Feminine, Androgynous, Alternating |  |
|  | Gender identity |  | Individual’s perception of their own gender, e.g. Male, Female, Non-binary |  |
|  | Preferred pronoun |  | Pronoun an individual chooses to identify with, and would prefer to use when talking to or about that individual, e.g. she, he, they, ze |  |
| BioPortal | Gender | Gender, Sex, and Sexual Orientation Ontology | Affirmed gender, Assigned gender, Assumed gender, authentic gender, felt gender, legal gender, natal gender | Ac22 |
|  | Affirmed Gender |  | Affirmed female, Affirmed male |  |
|  | Assigned Gender |  | Assigned female at birth, Assigned male at birth |  |
| AIHW | Gender | METeOR | 1-Male, 2-Female, 3-Other, 9-Not stated/inadequately described; alternate scheme M-Male, F-Female, X-Other | Ac23 |
| LOINC | Gender | LOINC, Version 2.65 | 77691-5 Gender Identity | Ac25 |
| SNOMED CT | Gender | SNOMED CT, 2020-03-09 Release | Examples only: 263495000\|Gender\|, 365873007\|Gender finding\|, 2851160012\|Gender identity finding\|, 772004004\|Non-binary gender\|, 703118005\|Feminine gender\|, 703117000\|Masculine gender\|, 248153007\|Male\|, 248152002\|Female\|,  407377005\|Female-to-Male (FTM)/Transgender Male/ Trans Man\|, 407376001\|Male-to-Female (MTF)/ Transgender Female/Trans Woman\|, 446131000124102\|Identifies as non-confirming gender (US synonyms include –Genderqueer; identifies as neither exclusively male nor female, non-binary gender)\| | Ac26 |

**Legends:** HL7-Health Level Seven, FHIR-Fast Healthcare Interoperability Resources, DICOM-Diagnostic Imaging & Communication, ONC ISA-Office of National Coordinator Interoperability Standards Advisory, NHS-National Health Services, ISO-International Standards Organization, AIHW-Australia Institute of Health and Welfare, LOINC-Logical Observations Identifiers Names and Codes, SNOMED CT-SNOMED Clinical Terms
